# Resilience in the Perinatal Period and Early Motherhood: A Principle-Based Concept Analysis

**DOI:** 10.3390/ijerph19084754

**Published:** 2022-04-14

**Authors:** Susan Elizabeth Hannon, Déirdre Daly, Agnes Higgins

**Affiliations:** School of Nursing and Midwifery, Trinity College Dublin, The University of Dublin, 24 D’Olier Street, D02 T283 Dublin, Ireland; dalyd8@tcd.ie (D.D.); ahiggins@tcd.ie (A.H.)

**Keywords:** resilience, mental health, perinatal, early motherhood, principle-based concept analysis

## Abstract

A context-specific delineation of research approaches to resilience in the perinatal and early motherhood literature is currently lacking. A principle-based concept analysis was used to establish a description of how women’s resilience is currently conceptualised and operationalised within empirical research in the perinatal period and early motherhood (defined as up to five-years postpartum). CINAHL, Medline, PsychInfo, EMBASE, ASSIA, Web of Science, Scielo, Maternity and Infant Care, the Cochrane Library, and the World Health Organization were systematically searched (January/February 2020 and March 2022). Fifty-six studies met the inclusion criteria. Analysis demonstrated interchangeable use of associated concepts such as ‘coping’, ‘coping strategies’, and ‘adaptation’. Resilience was frequently operationalised as the absence of illness symptomatology, rather than the presence of mental well-being. Investigations of positive areas of functioning were predominately related to the mother’s family role. There was limited qualitative exploration of women’s perspectives. Recommendations for the pragmatic application of resilience research were not well developed. The narrow operationalisation of resilience by mental ill-health and parental role, and the distinct absence of women’s perspectives, restricts the logical maturity and pragmatic application of the concept. Future research may benefit from exploration of women’s insights on indicators that might best reflect positive functioning and resilience in this period.

## 1. Introduction

Positive aspects of mental health and well-being are receiving increased research interest [1], particularly as there has been growing recognition that broader inquiry may advance our understanding beyond the contested conceptualisation of mental health as consisting of either illness or the absence of illness [2]. This shift also extends to the perinatal mental health literature [3]. Perinatal mental health research has typically focused on risk factors and negative outcomes, rather than on investigation from a positive or strengths-based perspective [4]. However, a growing body of research is emerging on women’s psychological health and resilience, and it seems that this interest is driven, at least in part, by the growing recognition that supporting mothers also supports families and improves outcomes for all [5,6].

The concept of resilience has been promoted as a perspective on health and well-being, which assists in shifting research foci away from ‘deficit’ models of illness and psychopathology [7,8], towards a better understanding of the processes, assets, or protective factors that enable mental health to be regained or maintained despite adversity [9].

Resilience is a debated topic within the literature and has been the subject of several discursive reviews, critiques, and analyses [7,10,11,12,13,14,15,16,17,18,19]. Some discussions have aimed to bring clarity to the concept independently of contextual elements [20], while others have endeavoured to explore the application of resilience within a specific context, such as in populations with HIV/AIDs [21], in adolescence [22], and mental health settings [23].

Scholars routinely draw attention to the on-going debate concerning the concept’s various definitions, the range of methods through which resilience is measured, and remark upon the consequent challenges to research synthesis and evaluation [15,24,25]. Definitions of resilience are often broadly grouped as taking trait or process perspectives.

Resilience research is, however, not amendable to discrete categorisation. Even within these perspectives, there is nuance and fluidity in the use of terminology, methodology and operationalisation, which makes categorisation in any context a challenge.

For example, Fletcher and Sarkar state that resilience perspectives may be organised by ‘trait, process, or outcome’ [15] (p. 3). However, references to various ‘outcomes’ of resilience are used with both ‘trait’ and ‘process’ orientations.

Trait conceptualisations regard resilience as a stable characteristic within an individual, whereas process conceptualisations consider the interacting influence of biological, psychological, social, cultural, and contextual factors on the individual’s experience [9,16,26]. Although there is less theoretical writing on resilience as an ‘outcome orientation’ within the literature, it has been described as maintaining or regaining mental wellbeing following a stressor, with the consideration that resilience is modifiable and influenced by factors that may protect against negative outcomes [24]. This is a description that echoes ‘process’ delineations.

Not only do differing conceptualisations influence researchers’ strategies for studying resilience, resilience is researched differently by context [24]. How resilience is operationalised in one particular circumstance may not be helpful or appropriate in another [9]. Thus, consolidating research within a specific context may allow researchers to identify recurring themes, strengths, and weaknesses within the literature and establish areas in which the concept has been well developed, or requires improvement.

This concept analysis aims to evaluate how women’s resilience is currently defined, conceptualised, and researched within the empirical literature, as it occurs in relation to the perinatal period and early motherhood, and to consider the concept’s degree of maturity, utilising Penrod and Hupcey’s [25] principle-based approach. The strength of this approach is that it uses scientific literature as data to reveal the ‘existing state of the science’ [25] (p. 403), to establish how the concept is currently used and conceptualised within the research according to the principles of epistemology, linguistics, pragmatism, and logic. Several concept analysis frameworks involve the identification of attributes frequently associated with the concept, for example, Walker and Avant [26], Rodgers [27], and Rodgers and Knafl [28]. However, such frameworks were considered unsuited to the aim of the current investigation, as attributes which appear to emerge from the analyses may be a result of frequently used measures.

In contrast to other styles of concept analysis, Penrod and Hupcey’s [25] methodology does not require the researchers to produce hypothetical cases to exemplify the concept. This feature of the framework was considered appropriate to the research aim of capturing the state of the science and avoids a researcher-produced interpretation of the manifestations of resilience.

## 2. Materials and Methods

### 2.1. Data Sources and Search Strategy

A protocol and search strategy for the concept analysis was developed a priori. The electronic bibliographic databases of CINAHL, Medline, PsychInfo, EMBASE, ASSIA, Web of Science, Scielo, Maternity and Infant Care, the Cochrane Library, and the World Health Organization (WHO) were systematically searched. As the population of interest was women in the perinatal period and the first five years postpartum, keywords for the first concept were (pregnan *) or ‘pregnant wom *’ or primigravid * or primipara * or ‘gravid *’ or multigravida * or multipara * or nullipara * or nulligravid * or childbearing or child-bearing or antenatal or ante-natal or prenatal or pre-natal * or ‘expect * mother *’ or perinatal * or peri-natal * or postnatal * or post-natal * or postpartum or post-partum * or ‘new mum *’ or maternal * or mother *. Keywords for the second concept, resilience (‘psychological resilience’/exp or resil *.) were combined using the Boolean operand ‘AND’. No date limit was applied, in order to capture all citations relevant to the analysis and identify development of the concept over time. Data were collected in January/February 2020 and March 2022.

### 2.2. Inclusion Criteria

Primary research published in English, where there was a clear expression that at least one of the phenomena examined or found was psychological/mental resilience of pregnant women and mothers up to five years postpartum. Research involving mothers and partners were included only where mothers’ data could be separated from partner data.

### 2.3. Exclusion Criteria

Articles were excluded if (i) resilience was examined during pregnancy only, (ii) if mothers’ resilience was explored in relation to infertility, miscarriage, stillbirth, or a child’s death, or (iii) if mothers’ resilience was operationalised by child health or development outcomes; and (iv) to avoid skewing the analysis, resilience research conducted with adolescent mothers was excluded. Adolescent motherhood entails period-specific challenges and navigation of dual adolescent and maternal identities [29], which women who become mothers in adulthood do not confront. In addition, education-related outcomes are common in adolescent resilience literature [22] and unlikely to feature as a life-stage appropriate domain of investigation for most mothers in adulthood. Articles evaluating interventions, or participant’s satisfaction with resilience interventions, conference abstracts, case studies, theses, reviews, and editorials were excluded; as were animal studies, non-English articles, and studies related to immunology or physical health.

### 2.4. Data Analysis

Articles were analysed following Penrod and Hupcey’s [25] principle-based concept analysis, which involved evaluation of data according to four philosophical principles: epistemology, linguistics, pragmatism, and logic. Penrod and Hupcey [25] acknowledge that concept analysis involves a degree of subjective interpretation, principles are not mutually exclusive, and points of interest that emerge in one principle may simultaneously hold relevance in another.

A data extraction tool adapted from O’Malley et al. [30] was used. Data included lead author’s discipline, study design, aim, sample characteristics, and country of origin. The tool also contained questions pertaining to the four philosophical principles. Each researcher (S.E.H., D.D., A.H.) analysed three studies independently using the data extraction tool and then discussed points of consistency and divergence within and between analyses. The researchers agreed on minor amendments to the extraction tool, and one researcher (S.E.H.) analysed the remaining studies.

The analysis did not entail a quality assessment of the included papers. The aim of the analysis was not to identify and synthesize findings from the individual papers, but to identify and evaluate the predominant methodological and philosophical approaches within the literature in this context.

## 3. Results

**Figure 1:**
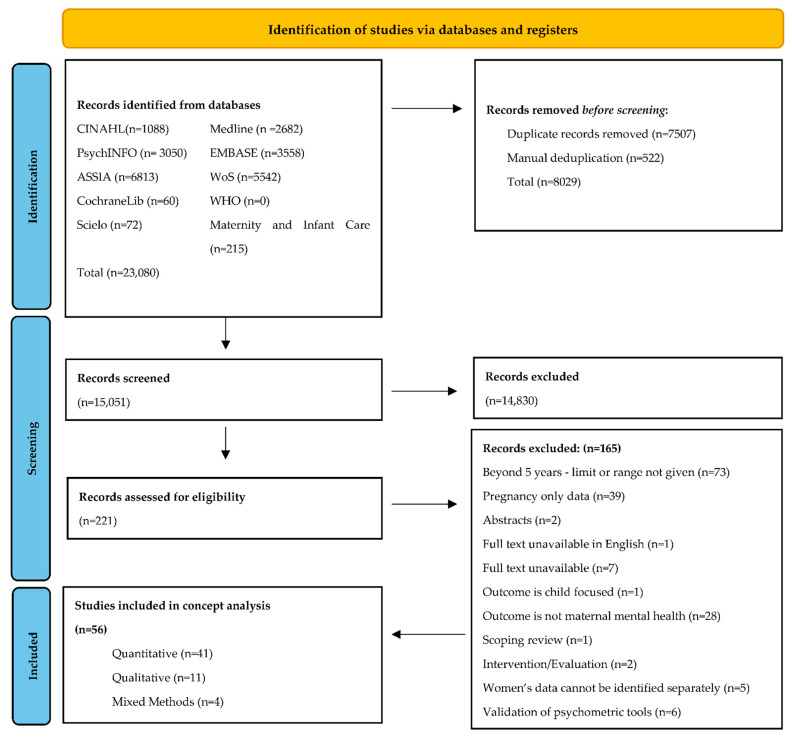
Prisma 2020 flow chart (adapted from Page et al., 2021) [31].

A total of 23,080 citations were retrieved, with 15,051 citations following duplicate removal. Title and abstract screening removed 14,830 citations, leaving 221 articles for full-text screening, 164 of which did not meet the inclusion criteria. A total of 56 studies were included for data extraction and analysis (Figure 1) [31].

Data were extracted from 41 quantitative, 11 qualitative, and four mixed-methods design studies, conducted since 2004. Approximately half of the included studies were published before the year 2020, and averaged at two per year prior to this date. The year 2020 saw a significant increase in research interest in resilience in the perinatal period and early motherhood, with eight studies published in 2020, 17 in 2021, and three in 2022. However, only eight of the 28 studies published since March 2020 included data from perinatal women living through the COVID-19 pandemic [32,33,34,35,36,37,38,39].

### 3.1. Epistemological Principle: Key Findings

The epistemological principle considers how a concept is defined and made distinct from other concepts within the literature [25]. An analysis of the included studies from the epistemological principle necessitates a consideration of two issues: (i) the provision and orientation of a formal definition of resilience, and (ii) how resilience is conceived by individual studies through review of the operationalisation and theoretical discussion of the concept. Of the 56 studies, 20 did not include any definition. Thirty-four studies formally defined resilience, of which 22 provided definitions that were from a trait perspective, and ten provided a process definition. Two studies gave an operational definition of resilience. Categorisations of definitions were determined by the explicit definition provided by the authors of each paper. (Table 1). Although orientations of resilience have been described as ‘trait, process, or outcome’ [15] (p. 3). A review of the theoretical discussion and operationalisations of resilience in the perinatal and early motherhood literature demonstrates that, overall, resilience was conceptualised in three ways: as a trait, which in some cases was simultaneously considered a protective factor; a process evidenced in mental health or well-being outcomes; and/or a trajectory exemplified by temporal patterns of low symptomatology.

Nineteen quantitative [35,37,40,41,42,43,44,45,46,47,48,49,50,51,52,53,54,55,56], two mixed-method [32,34], and one qualitative study [57] provided a definition which positioned resilience as a trait or ability. Adopting this perspective means resilience may also be regarded as a protective factor against negative outcomes, as demonstrated by Angeles García-León et al. [40], who used the Connor–Davidson resilience scale (CD-RISC) to explore resilience as a trait that is protective against pregnancy-specific stress.

Five quantitative studies [58,59,60,61,62] and five qualitative studies [63,64,65,66] conceived of resilience as a dynamic process, in which ‘psychological, social, environmental and biological factors interact to make an individual, at any stage of life, develop, maintain or regain their mental health despite exposure to adversity’ [67] (p. 1). Two studies offered multiple definitions, from both trait and process perspectives [45,68].

Resilience was researched by four studies as a pattern or trajectory of mental health outcomes evident in longitudinal data [69,70,71,72], each of these studies identified four temporal trajectories in mental health outcomes. These trajectories were determined as symptomology absence [71,72], or low scores of depressive symptomatology [70] and high quality of life (QoL) [69]. Denckla et al. [70] did not provide a conceptual or operational definition of resilience. However, Fonseca et al. operationally defined a resilient trajectory as demonstrated by the ‘maintenance of healthy adjustment over time, without disruption of functioning’ [69] (p. 113), similarly Kikuchi et al. defined resilience as women who were ‘not depressed throughout 1 year postpartum’ [71] (p. 632).

Twenty studies did not provide a formal conceptual definition of resilience. Within the 13 quantitative and two mixed methods studies, this left the reader to deduce how resilience was perceived, through consideration of the study’s methodological approach. For example, as a trait or ability, as would be suggested by the use of validated resilience scales [33,36,38,73,74]; as a protective factor [74,75,76,77,78]; as an outcome of low depressive [77,79]; or PTSD symptoms [80]; or as previously mentioned, a trajectory of low depressive symptoms [70]. Among the five qualitative studies that did not provide a definition, resilience was typically presented in relation to descriptions of coping [81,82,83,84], or through the identification of resiliency or protective factors [85,86].

#### Definitional Elements

The analysis found that the term ‘maternal resilience’ was used in six of the 56 studies [40,41,44,58,63,87]. However, none of the studies using this term provided a specified definition. It appears the authors’ use of ‘maternal resilience’ was intended to connect the concept of resilience to the context of the perinatal period and motherhood, rather than a term denoting some qualities or facets of resilience that are distinct to this period of life.

**Table 1:** Summary of Study Characteristics.

	**Quantitative Designs**		**Resilience Operationalised**						
				**Mental Ill-Health**					
**Author, Discipline**	**Country, Characteristics of Sample**	**Resilience Definition**	**Resilience Scales**	**Depression**	**Anxiety**	**Stress**	**PTSD**	**Other**	**Well-Being or Positive Functioning**
Andersson et al. (2021) [73] Computer Science.	Sweden. 4313 postpartum women from a population-based prospective cohort study. Data collected at 6 weeks postpartum (PP).	No formal definition	Resilience Scale for Adults (RSA) [88]	X	X			X	Sense of Coherence
Angeles García-León et al. (2019) [40] Psychology.	Spain. 151 pregnant women with low-risk pregnancy. Data collected in third trimester and approximately 15 days PP.	Trait/ability	Spanish translation of the CD-RISC (CD-RISC-10) [89]	X		X		X	Psychological Well-being
Asif et al. (2020) [49] Medicine.	Sweden. Sub-sample (n = 2026/6478) women. Data collected at 17 and 32 weeks gestation and 6 weeks PP.	Trait/ability	Resilience was operationalised by the sense of coherence scale (SOC) [90]	X					
Assal-Zrike et al. (2021) [79] Psychology.	Israel. Fifty-seven mothers of full-term infants and 48 mothers of preterm infants. Mothers were ethnic minority Bedouin-Arabs living in Israel. Data collected at 12 months PP.	No formal definition	Investigate the role of social support as a resilience factor for reduced postpartum emotional distress.	X	X			X	
Asunción et al. (2016) [75] Psychology.	Mexico. 280 low-income Mexican mothers aged ≥20 years. Data collected in pregnancy (>26 weeks) and at 6 weeks and 6 months PP.	No formal definition	Resilience Inventory (RESI) [91]	X	X			X	
Bennett et al. (2018) [41] Human Nutrition.	Ireland. 270 Irish and British women giving birth in Ireland. Data collected in pregnancy (>24 weeks) and at 17 weeks PP.	Trait/ability	Resilience Scale for Adults (RSA) [88]					X	* Maternal Well-Being
Chasson et al. (2021) [50] Social Work.	Israel. 152 first-time Israeli mothers, whose children were no older than two years old; 76 were single mothers by choice, and 76 were in a couple relationship.	Trait/ability	Brief Resilience Scale (BRS) [92]					X	Posttraumatic Growth
Denckla et al. (2018) [70] Public Health.	England. Data available from 12,121 women at two points during pregnancy and at 8 months and 2, 3, and 5 years PP.	No formal definition	Resilience was operationalised as a trajectory of stable, low levels of depressive symptoms.	X					
Fonseca et al. (2014) [69] Psychology.	Portugal. 43 couples (43 mothers and 36 fathers), aged ≥18 years, literate, with an infant diagnosed with a congenital abnormality (CA). Data collected at time of CA diagnosis and 6 months after the childbirth.	Operational definition: ‘Maintenance of healthy adjustment over time, without disruption of functioning’ (p. 113)	Resilience was operationalised as low psychological distress and high quality of life.					X	Quality of Life
Gagnon et al. (2013) [58] Epidemiology and Public Health.	Canada. 16 international migrant women (aged 27–38 years) participants had high psychosocial risk (low income, experience of violence, war or trauma, physical abuse). Data collected between 1 week and 4 months PP.	Dynamic process	Resilience was operationalised as low depression, no symptoms of anxiety/somatization or PTSD.	X	X		X		
Gerstein et al. (2009) [59] Psychology.	USA.115 families with a child with an intellectual disability between three and five years of age.	Dynamic process	Effects of parental wellbeing, marital adjustment, parent-child interaction (resilience factors) on trajectories of daily parenting stress (resilience outcome).			X			* Parental Well-Being
Grote et al. (2007) [60] Psychology.	USA. 179 married first-time parents. Data collected at five months of pregnancy and 6 and 12 months PP.	Dynamic process	‘Risk and resilience’ theoretical framework to examine the degree to which optimism (resilience factor) conferred protection against PPD (resilience outcome).	X		X			
Hain et al. (2016) [93] Psychology.	Germany. 297 women (aged 20–45 years). Data collected in the third trimester of pregnancy and at 6 and 12 weeks PP.	Both trait and process definitions	The RS-11 (Resilienzskala) [94]	X	X	X		X	
Handelzalts et al. (2020) [76] Psychology.	USA. Subset (n = 108/268) of women recruited from a longitudinal study oversampled for women who reported childhood abuse. Data collected at 4, 6, 12, and 15 months PP.	No formal definition	Religiosity and spirituality as resiliency factors for positive postpartum adjustment (resilience outcome) defined as low depression and high QoL.	X					Maternal Quality of Life
Harville et al. (2010) [80] Epidemiology.	USA. 295 pregnant women (222 completed) and 365 postpartum (eight weeks) women (292 completed) living in Louisiana who were exposed to Hurricane Katharina.	No formal definition	Resilience was operationalised as low depression and low/no PTSD.	X			X		Perceived Benefits:Personal Growth (single item)
Harville et al. (2011) [42] Epidemiology.	USA. 365 mothers exposed to multiple disasters. Data collected via phone interview at 2 months PP and survey questionnaire at 12 months PP.	Trait/ability	Brief Resilience Scale (BRS) [92]	X			X		Perceived Benefit
Julian et al. (2021) [51] Psychology.	USA. 233 ethnically diverse women from a prospective longitudinal study. Resilience resource data were collected during pregnancy and depressive symptoms were assessed between 4 to 8 weeks PP	Trait/ability	Moderating role of mastery, dispositional optimism, and spirituality (resilience resources) against the impact of stressful life events occurring in pregnancy and subsequent symptoms of PPD.	X					
Kikuchi et al. (2021) [71] Psychiatry.	Japan. Sub-sample (n = 11, 668/22,493) women. Women were recruited in pregnancy and depressive symptoms assessed at 1 month and 1 year PP.	Operational definition: ‘not depressed throughout 1 year postpartum’. (p. 632)	Resilience was operationalised as a trajectory of depressive symptomology absence.	X					
Ladekarl et al. (2021) [35] Obstetrics and Gynaecology.	Denmark. 73 women enrolled during pregnancy before (n = 26) and during (n = 47) the COVID-19 pandemic. Data were collected in the second trimester and at two months PP.	Trait/ability	Connor-Davidson Resilience Scale (CD-RISC) [95]	X	X	X			
Liu et al. (2020) [36] Mental Health.	USA. 506 postpartum women taking part in the PEACE (Perinatal Experiences and COVID-19 Effects) study. Data were collected online within 6 months PP.	No formal definition	Connor–Davidson Resilience Scale (CD-RISC) [95]	X	X		X	X	
Margalit et al. (2006) [43] Psychology.	Israel. 70 mothers from ‘intact families’ with infants aged 2–39 months and diagnosed as at-risk for delayed development.	Trait/ability	Resilience was operationalised using the sense of coherence scale (SOC) [90]			X			Family Adaptability and Cohesion, Coping
Martinez-Torteya et al. (2018) [44] Psychology.	USA. Sub-sample (n = 131/256) of women from a longitudinal study over sampled for women who reported childhood abuse. Data collected at 4 and 6 months PP.	Trait/ability	Connor–Davidson Resilience Scale (CD-RISC) [95]	X					Parenting Sense of Competence
Mautner et al. (2013) [45] Psychology.	Austria. 67 women German-speaking women who were diagnosed with preeclampsia in a previous pregnancy, and who gave birth within the last four years.	Trait/ability	The RS-13 [96]	X			X		Health Related Quality of Life
McNaughton Reyes et al. (2020). [78] Health Behaviour.	South Africa. 1480 pregnant women who recently became aware of their HIV positive status in South Africa. Participants were recruited in pregnancy and data collected at 14 weeks and 9 months PP.	No formal definition	Moderating role of socio-economic status, family social support, religiosity, or a vulnerability effect: baseline distress, childhood abuse history, HIV diagnosis (resiliency factors) on the long-term impact of physical/sexual IPV exposure and subsequent postpartum distress.					X	
Mikuš et al. (2021) [52] Obstetrics and Gynecology.	Croatia. 227 puerperal women giving birth in Croatia. Data collected on day 3 PP.	Trait/ability	Connor–Davidson Resilience Scale (CD-RISC) [95]					X	
Miranda et al. (2012) [77] Psychology.	Brazil. 52 women with low socioeconomic status who experienced a preterm birth 6–12 months prior to the study.	No formal definition	Resilience was operationalised as low depressive symptoms and/or low PPD.	X					
Mitchell et al. (2011) [74] Social Science.	USA. 209 African American mothers (aged 21–45 years) of varying socioeconomic status, whose babies were two to 18 months old.	No formal definition	Connor–Davidson Resilience Scale (CD-RISC) [95]	X	X				
Mollard et al. (2021) [37] Nursing.	USA. 885 women who gave birth in the USA during the first wave of the COVID-19 pandemic in the USA.	Trait/ability	Connor–Davidson Resilience Scale (CD-RISC) [95]			X			Mastery
Monteiro et al. (2020) [62] Psychology.	Portugal. 661 postpartum women with infants between 0 and 12 months.	Dynamic process	Resilience Scale for Adults (RSA) [88]	X					Mental Wellbeing, Maternal Confidence, Self-Compassion, Psychological Flexibility
Muzik et al. (2016) [56] Psychiatry.	USA. Sub-sample (n = 116/256) of women from a longitudinal study over sampled for women who reported childhood abuse. Data collected at 4, 6, and 18 months PP.	Trait/ability	Connor–Davidson Resilience Scale (CD-RISC) [95]					X	
Nishi et al. (2017) [46] Psychiatry.	Japan. 117 women (aged ≥20 years), Japanese speaking, and literate, recruited in pregnancy at 12–24 weeks gestation and assessment follow-up completed at 4 weeks PP.	Trait/ability	Tachikawa Resilience Scale (TRS) [97]	X					Post Traumatic Growth
Perez et al. (2021) [72] Psychology.	USA. 70 mothers and 50 fathers, (data were separable) of a child diagnosed with a disorder/difference of sex development (DSD). Participants were recruited when their child was <2 years old. Data were collected prior to a child receiving genitoplasty, and at 6 and 12 months post-surgery.	No formal definition	Resilience was operationalised as a trajectory of ‘consistently low levels of (depression) symptoms across time.’ (p. 589).	X					
Puertas-Gonzalez et al. (2021) [38] Psychology.	Spain. 212 participants; 96 gave birth before the COVID-19 pandemic and 116 during the COVID-19 pandemic. Data were collected one month PP.	No formal definition	Connor–Davidson Resilience Scale (CD-RISC) [95]	X		X		X	
Sahin (2022) [53] Psychiatry.	Turkey. 120 women recruited in pregnancy. 120 completed assessment during pregnancy, and 77 women completed assessment one month PP.	Trait/ability	Connor–Davidson Resilience Scale (CD-RISC) [95]	X				X	Maternal Attachment
Schachman et al. (2013). [61] Psychology.	USA. 71 women married to (but were not themselves active-duty service women) active-duty military members stationed at a USA military base, delivered a singleton live baby within 3 months of the study.	Dynamic process	Effects of family changes and strains, self-reliance, social support (protective factors) on postpartum depression (outcome).	X					Family Changes and Strains, Self-Reliance, Social Support
Sexton et al. (2016) [47] Psychology.	USA. Sub-sample (n = 141/256) of women from a longitudinal study over sampled for women who reported childhood abuse. Data collected at 4 months PP.	Trait/ability	Connor–Davidson Resilience Scale (CD-RISC) [95]	X			X		Family Specific Well-Being, Postpartum Mastery
Sexton et al. (2015) [48] Psychology.	USA. Sub-sample (n = 214/256) of women from a longitudinal study over sampled for women who reported childhood abuse. Data collected at 4 months PP.	Trait/ability	Connor–Davidson Resilience Scale (CD-RISC) [95]	X			X		Family Functioning, Postpartum Sense of Competence
Verstraeten et al. (2021) [68] Obstetrics and Gynecology.	Canada. 200 women who experienced a wildfire in Canada during, or shortly before, pregnancy. Women were recruited within one year of the wildfire.	Both trait and process definitions	Connor–Davidson Resilience Scale (CD-RISC) [95]				X	X	
Werchan et al. (2022) [39] Cognitive Science.	USA. Data collected during the COVID-19 pandemic from 4412 pregnant and postpartum (within first 12 PP months) women used to identify risk and protective/resiliency factors associate with four behavioural coping phenotype profiles.	No formal definition	Research identified coping phenotypes or profiles associated with risk and resiliency for adverse mental and physical health outcomes.	X	X			X	
Yu et al. (2020) [54] Public Health.	China. 1126 women recruited in pregnancy from two urban maternal and child health hospitals in Hunan province, China. Data were collected at four time points (3 times during pregnancy and at 6 weeks PP).	Trait/ability	Brief Resilience Scale (BRS) [92]	X	X			X	
Zhang et al. (2021) [55] Gynecology and Obstetrics.	China. 200 pregnant women admitted to hospital for preterm labour. Postpartum PTSD was evaluated at 6 weeks PP.	Trait/ability	Connor–Davidson Resilience Scale (CD-RISC) [95]			X		X	
**Mixed-Methods Designs**			**Resilience Operationalised**						
**Author, Discipline**	**Country, Characteristics of Sample**	**Resilience Definition**	**Resilience Scales**	**Depression**	**Anxiety**	**Stress**	**PTSD**	**Other**	**Well-Being or Positive Functioning**
Davis et al. (2021) [32] Mental Health.	Australia. Sub-sample (n = 174/461) of perinatal women living through the COVID-19 pandemic in 2020.	Trait/ability	Resilience was operationalised through scales measuring mindfulness and self-compassion.			X			Mental Well-being
	A stratified sub-sample (n = 14/174) completed the qualitative component.		Qualitative Findings: Interviews conducted with seven women from the ‘high’ resilience group and seven from the ‘low’ resilience group. Both groups identified the social, emotional, psychological, healthcare service, and informational needs of perinatal women during the COVID-19 pandemic.						
Farewell et al. (2020) [33] Health and Behavioural	USA. 31 pregnant and postpartum women (within 6 months PP), living in Colorado, during the COVID-19 pandemic.	No formal definition	Brief Resilience Scale (BRS) [92]	X	X			X	Mental Well-being
Sciences.			Qualitative Findings: Sources of resilience identified by participants included using virtual communication platforms, having positive partner emotional support, being outdoors, focusing on gratitude, setting daily routines, and self-care behaviours, such as engaging in physical activity, getting adequate sleep and eating well.						
Kinser et al. (2021). [34] Nursing.	USA. Mixed-methods research with 524 pregnant and postpartum (up to 6 months PP) women. Data were collected	Trait/ability	Connor–Davidson Resilience Scale (CD-RISC) [95]	X	X		X		
	during the early stages of the COVID-19 pandemic.		Qualitative Findings: Adaptability and resilience building activities were defined as: taking time to get outdoors, getting exercise and eating well, use of mindfulness practices and meditation, use of prayer, using social media for connection with family and friends, and accepting help.						
Edge and Roger (2005). [81] Epidemiology.	England. Theoretic sampling of 12, inner city, Black-Caribbean women for in-depth interviews at 6–12 months PP.	No formal definition	The authors presented resilience under the narrative of ‘Strong-Black-Women’. An identity theme characterised by an active resistance to symptomatology and labelling, with resilience being linked to coping and problem solving. Quantitative data were not reported.						
	**Qualitative Designs**		**Resilience Operationalised**						
**Author, Discipline**	**Country, Characteristics of Sample**	**Resilience Definition**							
Farewell et al. (2021). [85] Health and Behavioural Sciences.	New Zealand. 74 mothers of children under the age of five, living in a high deprivation neighbourhood in Auckland, NZ. Data were collected via one-to-one interviews and focus groups.	No formal definition	‘Protective factors’ and ‘resources’ were presented as promoting resilience/positive mental health and well-being in this research. The researchers developed a priori codes hypothesised to promote resilience among mothers across ethnic groups. Themes linked to socioecological resources that support positive mental health and well-being included: (1) social support: support from family and friends offering emotional and instrumental support. (2) community level: neighbourhood cohesion, community involvement, community resources. (3) societal-level factors: cultural identity and alignment with social and cultural norms.						
Gewalt et al. (2018). [82] Public Health.	Germany. Nine asylum-seeking women (aged 22–37 years) living in state provided accommodation. Interview data collected at two points during pregnancy and at 6 weeks PP.	No formal definition	Authors interpret social support and coping styles as factors that increase resilience and act as counterbalances to psychosocial stressors.						
Goodman et al. (2020). [65] Obstetrics and Gynecology.	USA. Ten women in New England who had entered treatment for opioid use disorder during pregnancy, and engaged in treatment in the postpartum period. Data were collected in interviews between 2 weeks and 1 year PP.	Dynamic process	Within data collected in semi-structured interviews with women with opioid use disorder, who continued to engage in treatment during the postpartum period, the theme of resilience was identified by the researchers as emerging and developing as an adaptive and dynamic process. Resilience was considered evident through complex interactions between individual-level inner motivations and self-efficacy, and women’s abilities to positively utilise external resources such as engagement with clinicians and peers.						
Keating-Lefler and Wilson. (2004). [57] Nursing Science.	USA. 20 single, first time mothers, Medicaid-eligible, and living in poverty. Recruited in pregnancy and interviewed at 1, 2, and 3 months PP. Aged ≥19 years, English-speaking.	Trait/ability	Authors position qualitative findings within a grief framework; resilience was considered integral to the negotiation of ‘multiple losses’ experienced by un-partnered mothers, and held within the theme of ‘reformulating life’.						
Keating-Lefler et al. (2004). [84] Nursing Science.	USA. 5 single mothers with and infant less than 1 year, low income, not living with child’s father, and attending a women, infants, and children clinic.	No formal definition	Resilience was a subtheme of ‘transition’, though resilience and its attributes were undefined by this study.						
Nuyts et al. (2021). [87] Midwifery/ Epidemiology.	Belgium. Purposive sample of 13 women without pre-existing bipolar and psychotic disorders or a depressive or anxiety disorder, admitted to an infant mental health outpatient service in Belgium when their infant was aged 1 to 24 months.	Dynamic process	Data concerned the professional support needs of mothers prior to admission to an infant mental health day clinic. The three themes identified were ‘experience of pregnancy, birth, and parenthood’; ‘difficult care paths’; and ‘needs and their fulfilment’. The theme ‘experience of pregnancy, birth, and parenthood’ contained three subthemes: (1) ‘reality does not meet expectations’, (2) ‘resilience under pressure’, and (3) ‘despair’. The theme ‘resilience under pressure’ was not developed, and the term resilience appeared interchangeable with ‘mental health’.						
Rossman et al. (2016). [63] Nursing Science.	USA. Socio-economic and ethnically diverse subsample (n = 23/69) of mothers of very-low birth weight infants derived from a study on maternal role attainment. Qualitative interview data collected between 4 and 8 weeks PP.	Dynamic process	Characteristics considered demonstrative of resilience were mothers using resources to actively promote their mental health, reframing or redefining their lives, acceptance of reality, advocating for their infants, positive functioning in daily life, and envisioning the future.						
Schaefer et al. (2019) [64] Psychology.	USA. Racially diverse sample of 10, low-income women who experienced intimate partner violence (IPV) during or immediately prior to pregnancy and had given birth within the last year, and 46 service providers who interacted directly with women exposed to IPV in pregnancy.	Dynamic process	Authors identified the overarching theme of ‘strengths’, which was comprised of ‘transformation’ and ‘resilience’. ‘Strengths’ were understood as character traits possessed by pre- and postpartum mothers exposed to IPV around pregnancy. Resilience was considered demonstrated through women’s continued efforts to access individual resources and seek community support.						
Shadowen et al. (2022) [86] Obstetrics and Gynaecology.	USA. 8 postpartum women receiving medication for opioid use disorder. Data were collected between 2 and 6 months PP.	No formal definition	The researchers identified the theme of ‘building resilience amidst trauma and pain’ within the qualitative data provided by postpartum women receiving medication for opioid use disorder. ‘Building resilience’ was linked with themes of transformation and perseverance in overcoming traumatic experiences and stigma as part of their recovery journey.						
Shaikh et al. (2010) [83] Sociology.	Canada. 12 women (aged 24–39 years), residing in underserviced rural communities, with a psychiatric diagnosis of Postpartum Depression (PPD), or who self-identified as having suffered from PPD within one year after birth and no more than five years prior to the study.	No formal definition	Authors equated resilience with ‘coping strategies leading to successful adaptation or positive outcomes under stressful or adverse circumstances.’ (p. 3). Coping strategies were identified using four theoretical components: Existential philosophy: meaning making strategies; Cultural relational theory: seeking support; Feminist standpoint theory: nurturing oneself and advocacy work; Beyond theoretical framework: connecting with nature.						
Theodorah et al. (2021) [66] Nursing.	South Africa. Qualitative interviews with 10 first-time mothers within the first six months PP.	Dynamic process	Two themes and subthemes were identified: (1) ‘challenges, empowerment, support, and resilience during initiation of exclusive breastfeeding’ –subcategory: ‘support and resilience during early breastfeeding (EBF) initiation; (2) ‘diverse support and resilience during maintenance of exclusive breastfeeding’—subcategory: ‘support and resilience during EBF maintenance’. Differences between categories were not well specified and themes of resilience were not developed.						

Key: X = Study used one or more psychometric scale measuring depressive, anxiety, stress, or PTSD symptomatology, or other psychopathology; * Psychopathology tools used to measure ‘wellbeing’ or ‘positive function’.

### 3.2. Linguistic Principle: Key Findings

The linguistic principle evaluates if terminology is context appropriate and consistently used. There was evidence of fluidity in the use of resilience with associated terms and concepts, shifts in meaning or application of the concept varied across papers and it was challenging to disentangle linguistic and operationalised use; as such, linguistic consistency was not easily categorised.

There were several examples of coping or coping strategies used synonymously with resilience [54,58,81,82]. For Nishi and Usada, ‘stress-coping ability’ [46] (p. 3) was equated with resilience. Others considered coping a manifestation of resilience [83] or distinguished coping as an ‘attribute of resilience rather than a concept in its own right’ [63] (p. 435), while Werchan et al. [39] quantitatively categorised pregnant and postpartum women into four behavioural profiles associated with either ‘low’, ‘passive’, ‘active’, or ‘high’ coping styles, using depression, anxiety, global distress, and behavioural coping surveys. The terms ‘resistance’, ‘adaptation’, and ‘protection’ were a frequent feature of the literature, and occasionally used synonymously with resilience [34,53,56,60,76,77]. In these instances, resilience was evidenced in positive patterns of scoring on mental health scales, which were considered demonstrative of an adaptive response, or resistance to negative outcomes [49]. For example, Grote and Bledsoe, using a ‘risk and resilience’ theoretical framework [60] (p. 109), examined if optimism during pregnancy might impact on depression severity postpartum and interpreted the positive moderating effect of optimism on depressive symptoms as conferring resilience, protection, and resistance. The topic of adaptation in qualitative data was associated with self-care or activities that the authors considered as ‘resiliency building’, such as getting exercise and eating well, use of mindfulness practices and meditation, prayer, and accepting help [34].

Resilience research is considered to offer a divergence from ‘deficit’ models of illness and psychopathology [7] (p. 1). However, the use of ‘deficit’ language appeared, albeit infrequently. For example, Asunción et al. [75] when referring to Schachman and Lindsey’s [61] research findings, cited the absence of ‘self-resilience’ as meaning women who ‘lack a positive attitude, perseverance, self-efficacy, and the ability to adapt to the stress of having a new baby’ [75] (p. 831). However, Schachman and Lindsey did not employ language suggesting inadequacy but rather used positive terms, suggesting mothers met challenges with ‘self-reliant’ and ‘can-do’ attitudes [61] (p. 164). Deficit language appeared in Bennett and Kearney’s commentary that a personal commitment to breastfeeding may sustain women to ‘continue to breastfeed despite any shortcomings in their support network or resilience’ [41] (p. 8). While this phrasing may simply be an oversight rather than a judgment on women, the use of deficit language sits incongruously with resilience as a strengths-based approach. Additionally, the use of resilience scales was occasionally accompanied by the categorisation of women as possessing ‘low’, ‘high’ [35,52,53,73], and, in one case, ‘normal’ [49] resilience levels based on cut-off scoring.

### 3.3. Pragmatic Principle: Key Findings

The pragmatic principle focuses on how the concept has been used within the literature, whether it accurately describes the phenomenon, and its usefulness to clinical practice.

Forty-one studies used quantitative measures for resilience; 17 operationalised resilience through mental health outcomes and/or positive functioning [32,39,49,51,58,59,60,61,69,70,71,72,76,77,78,79,80], while 23 employed a resilience scale alongside one or more surrogate outcomes [35,36,37,38,40,41,42,44,45,46,47,48,50,52,53,54,55,56,62,68,73,74,75,93], and one operationalised the sense of coherence scale for resilience [43]. Among the 11 qualitative studies, five studies linked resilience to evidence of coping or coping strategies [63,64,81,82,83], and two to adaptation to difficult life circumstances [57,84].

#### 3.3.1. Operationalisation and Research Pragmatism

As in the wider literature, facets of mental health such as depression, anxiety, or stress were utilised as surrogate outcomes for resilience. In such cases, absent or low-level symptomatology were considered indicative of resilience. Scales developed specifically for use in the perinatal period and parenthood were employed to explore outcomes such as parenting stress [43,59], maternity blues [52], pregnancy pressure [55], pregnancy-specific stress [40], postpartum emotional distress [79], pregnancy-related anxiety [93], and pregnancy distress [41]. Other outcomes included post-traumatic stress disorder (PTSD) [34,36,42,45,47,48,55,58,68,80], suicidal ideation [56] or suicidal behaviour [75], psychopathological symptoms [38,40], anxiety [35,54,74,79], loneliness [33], and, most frequently, depression [35,42,44,45,47,48,49,54,60,61,62,70,72,73,74,75,76,77,79,80,93].

Although this approach is common within resilience literature as a whole, operationalising resilience as the absence of psychopathology has been criticised [98]; as the absence of illness does not necessarily indicate the presence of health or successful adaptation, which is considered a hallmark of resilience [99].

Two studies operationalised reports of perceived benefit following adverse life conditions as a positive outcome [42,80]. Seventeen of the quantitative studies operationalised positive domains of functioning to explore resilience beyond absent symptomatology. Positive function was regarded as high scores in QoL [45,69,76], psychological well-being [32,33,40], posttraumatic growth [46,50], postpartum mastery and family specific well-being [47], family adaptation [43], postpartum sense of competence [44,47], sense of coherence [73], self-compassion and mindfulness [32], mastery [37], flourishing, maternal confidence, self-compassion, psychological flexibility [62], and maternal attachment [53].

However, two studies, which intended to capture evidence of a well-being component of resilience applied scales designed to measure psychopathology. For example, Bennett and Kearney [41] used the mother and baby interaction scale (MABISC) [100], which was developed to assess maternal distress and suboptimal mother-infant bonding rather than maternal well-being, as was the authors’ intention. Likewise, Gerstein et al. [59] proposed to operationalise parental well-being through the Symptom Checklist-35 [101], though this scale measures symptomatic distress [102].

#### 3.3.2. Stakeholders’ Interpretations of Resilience in the Context of the Perinatal Period and Early Motherhood

Penrod and Hupcey state that the pragmatic maturity of a concept involves the members of the discipline recognising the ‘manifestations of the concept; it should ring true with experience’ [25] (p. 405). A salient question to ask then is ‘who has identified the manifestations of resilience in the perinatal period and early motherhood; researchers, healthcare professionals, or mothers?’

In five qualitative studies, resilience emerged inductively from researchers’ analysis of data. For example, Farewell et al.’s [85] socio-ecological investigation of protective resources available to mothers in areas of deprivation developed codes a priori, to link interpersonal supports, and community level and societal-level factors in promoting resilience/positive mental health and well-being. In other studies, authors concluded that resilience was manifested through the use of coping strategies among mothers with perinatal depression [81,83]. Others suggested that social support and coping style gave rise to resilience by counterbalancing psychosocial stressors [82].

In Rossman et al.’s [63] study, resilience was related to coping; however, the authors provided a rounded discussion on the frequent synonymous use of the term coping and resilience, and clearly stated their understanding of the functional difference between the two concepts. This study, though specific to the complexities of mothering an infant in NICU, emphasised the practical role that healthcare professionals may have as a resilience-promoting influence for women. This support was identified by women as being pivotal for their mental health and navigation of their unexpected circumstances and was interpreted by the researchers as enhancing resilience through nurturing a woman’s confidence in her capabilities as a mother.

Schaefer et al.’s [64] focus group data from both women participants and service providers offered insight on how resilience may be understood from different stakeholder perspectives. The resilience of women who were exposed to intimate partner violence during pregnancy was conceived as ‘utilizing resources to keep moving forward’ (p. 13). Data relating to perseverance, self-reliance, and reconnecting to community, which were described by the authors as resilience enhancing, were identified more frequently within data obtained from service providers than from narratives offered by mothers. This led the authors to suggest that women were less likely than service providers to identify their own strengths and the assets that are pivotal to resilience responses.

No study reported on women’s views of resilience and mental health in the perinatal period and motherhood. Although the interview schedule from one study contained the question ‘What does perinatal resilience mean to you?’, the findings from this question were not reported [87] (p. 3).

#### 3.3.3. Clinical Pragmatism:

Clinical pragmatism focuses on the utility of the concept within, and its potential to guide, clinical practice. Two themes evident in authors’ recommendations for the clinical application of their findings were that healthcare professionals should support and inform, and assess and screen.

Rossman et al. [63] highlighted breastfeeding peer counsellors’ and neonatal intensive care unit (NICU) nurses’ unique role as support sources for women, and detailed practical ways that nurses can foster resilience in mothers, such as promoting women’s sense of maternal self-efficacy through validating and normalising their experience. Bennett and Kearney advocated ‘supporting women to support themselves’ [41] (p. 609), and suggested that healthcare professionals offer support to women, in the form of breastfeeding education, to enable women to build their own supports independently. Two studies that linked successful coping with resilience proposed that women be informed of, and supported in, the use of coping strategies [81,83]. However, the practicalities of where, how, and by whom these recommendations might be implemented were not described.

Multiple authors recommended that screening or assessment of mothers could be a practical application of their findings [87,93]. Edge and Roger [81] drew attention to the need for perinatal mental health screening to be culturally sensitive. Muzik et al. [56] called for multiple points of contact and assessment for postpartum women, beyond the traditional six-week period; and Fonseca et al. (p. 120) [69] suggested that parents of infants with a congenital abnormality could benefit from comprehensive assessment of their ‘adjustment indicators’ using psychological distress and QoL tools. However, the varied ways in which resilience was conceptualised across the studies raises questions on how screening should be achieved, and a dialogue as to whom, how, when, and where they should be conducted was not elaborated upon. Questions on whether screening is best conducted using resilience scales or mental health tools did not emerge from the analysis. Additionally, ethical issues concerning consent and autonomy [103] and acceptability of screening procedures to women and healthcare professionals [104] were not addressed.

### 3.4. Logical Principle: Key Findings

The logical principle examines the theoretical integration of the concept in question with associated concepts. Within the 56 studies, concepts relating to mental health, QoL, adaptation and adjustment, coping, and coping strategies emerged alongside resilience.

#### 3.4.1. Mental Health

Several studies acknowledged that psychological outcomes should extend beyond the absence of psychopathology and contain measures intended to capture positive psychological or mental health outcomes. Angeles García-León et al. [40] employed a psychological well-being scale, and, though not situated directly as mental health, QoL was operationalised by three studies as a domain that may demonstrate a positive outcome of resilience [45,69,76]. Davis et al. [32] operationalised resilience through scales measuring mindfulness and self-compassion, and mental health as both stress and well-being; while, Monteiro et al. [62] explored the well-being aspect of resilience through scales for flourishing, maternal confidence, self-compassion and psychological flexibility. However, the most frequently utilised measures were those designed to measure mental distress/illness, such as anxiety, depression, or PTSD. This method of operationalisation risks the absence of mental distress/illness being conceived of as exemplifying resilience in the perinatal period and early motherhood.

#### 3.4.2. Adaptation and Adjustment

Adaptation and adjustment are perhaps expected within the context of motherhood, given that it is widely regarded as a transitory period [105]. However, conceptual boundaries between resilience and adaptation or adjustment were not always clear. For example, Fonseca et al. [69] operationalised parental adjustment through measures of psychological distress and QoL. Resilience was conceived as a trajectory of low distress and high QoL scores over time, such that ‘good’ parental adjustment was defined as the ‘resilient’ trajectory. In this way, parental adaptation and resilience were one and the same, without a linguistic or functional distinction being made by the authors. Similarly, Handelzalts et al. [76] operationalised positive postpartum adjustment as low depression and high QoL, interpreting the moderating effect of religiosity and spirituality on these measures as having a resilience-enhancing influence. Additionally, adaptation was typically aligned with measures of functioning that were specifically situated in the context of a mothering role, such as Schachman et al.’s [61] investigation of maternal role adaptation, Sexton et al.’s [47] use of family specific well-being and postpartum Mastery measures, Sahin’s [53] use of maternal attachment, and Sexton et al.’s [48] exploration of postpartum positive functioning.

#### 3.4.3. Coping

The degree to which coping was regarded as distinct from or integrated with resilience varied within the included studies. Several studies [39,46,83] conceived of coping as a manifestation or appropriate operationalisation of resilience, which may be considered an intentional blurring of concepts, as coping was considered to be held within resilience. Mikuš et al. explicitly equated stress coping ability with resilience and defined it as ‘inseparable from anxiety, depression and stress reactions’ [52] (p. 345).

Gagnon et al.’s [58] conceptualisation was vaguer, appearing to situate coping strategies somewhere between an attribute and an outcome. For others, resilience and coping were used interchangeably, and it would seem, though not discussed by the authors, that these were understood to be, if not synonymous, then held within the construct of the other [81,82].

Some authors investigated coping and coping strategies as attributes or associates of resilience [34,54]. Rossman et al. considered coping as an attribute of resilience and made a distinction between resilience as a contextually variable process ‘oriented toward positive outcomes’, while coping was understood as ‘the behaviour that follows the appraisal’ [63] (p. 435). The point of difference being that adaptive coping was related to resilience, in contrast to maladaptive coping mechanisms, which led to negative outcomes.

## 4. Discussion

Resilience has been widely researched in relation to periods of growth and transition, and there is a general acknowledgement that resilience can be attained or develop at any point in life. However, there has been little research in the context of the perinatal period and motherhood. This represents an exciting opportunity to develop and advance this research, and apply the lessons learned from the wider literature, as well as those emerging from the current analysis, in order to improve upon the clarity and consistency in this context. This concept analysis offers a timely analysis of common epistemological and methodological trends in resilience research with perinatal women and women in the early years of motherhood. Twenty-eight of the 56 included studies were published between 2004 and 2019, a 15-year timespan, while 28 were published in the last two years, between 2020 and 2022.

A challenge in evaluating consistency in the use of the term resilience, in the context of the perinatal period and motherhood, is that first, varying definitions and interchanging use of terms and concepts is an issue already remarked upon in relation to resilience in any context [15,106]. Second, authors’ conceptualisations of resilience shape how it is used linguistically and how it is researched practically and logically within individual studies.

Examining the contextual use of resilience unveiled some overlap between the epistemological and linguistic principles, as demonstrated by the use of the term ‘maternal resilience’. Though ‘maternal resilience’ was used within six studies, its use is potentially misleading. Epistemologically, it implies the existence or investigation of boundaries that establish distinct elements of resilience within the subject of maternity or motherhood. However, ‘maternal resilience’ was not employed to denote unique definitional or conceptual features of resilience as it occurs within the perinatal period and motherhood. Linguistically, it became apparent that the usage within the studies was simply to place the phenomenon of resilience within the context of the perinatal period and motherhood. Luthar et al. [9] encourage the use of terminology that links resilience to the context in which it is being researched, as doing so brings specificity to findings and demonstrates a clear boundary, which resilience, considered evident in the positive outcomes of one domain, does not imply resilience across domains. Researchers should be careful to specify the relevance of the areas or concepts used to operationalise resilience and be conscious that the outcomes in which resilience is explored are not global, but compartmentalised indications of resilience [18].

Although Nuyts et al. [87] utilised Van Haeken et al.’s definition of ‘perinatal resilience’ of mothers and partners in the first 1000 days of life as a ‘circular process towards greater well-being’ [107] (p. 1), specific definitional elements, which may be inimitable to this timeframe, did not emerge in the analysis of the included studies. This is perhaps a reflection of the emergent nature of the research in this context and an indication that the topic may benefit from further investigation and advancement.

Overall, definitions typically borrowed from conceptualisations found in the wider literature. This is not a criticism of the research concerning the perinatal period and motherhood, as the definitional conflicts of resilience are a matter of differing theoretical perspectives rather than context and will likely continue to be a matter of contention among theorists. However, the included studies seldom addressed the definitional issues and subsequent empirical consequences for the wider literature, or the implications for research in the perinatal period and motherhood.

Researchers should take care to address the relevant concerns surrounding the multifaceted interpretation of resilience as a concept and remain mindful of its limitations. One practical measure that researchers may take to improve conceptual clarity is to provide clear definitional and conceptual positions of resilience and its operationalisation within their research. Readers with an interest in resilience would benefit from researchers maintaining consistency between the conceptual definitions and methods used to operationalise resilience within their research. Furthermore, research may be improved through the provision of a clear exploration of the concepts and constructs employed within resilience research, and researchers should take care to demonstrate recognition of the capacity and limitations of tools designed to measure illness, in order to prevent ‘absence of illness’ being confused with the presence of health.

Additionally, a possible avenue for advancement may be through the inclusion of women’s perspectives regarding resilience in this context. Though data from several qualitative studies were included in the analyses, none specifically sought women’s views on how resilience may be defined or manifest in the perinatal period and early motherhood. Typically, resilience was not the primary concept of interest among the qualitative studies; rather it emerged in researchers’ analysis of data regarding mothers’ experience and responses to multiple adverse life circumstances. A more inclusive understanding of mothers’ perspectives on resilience and their insights into the factors leading to vulnerability and protection has the potential to inform the development of effective prevention and intervention strategies.

Concepts associated with resilience, such as coping appeared frequently, though with marked variability between studies with regards to whether coping was considered distinct from, or integrated with, resilience. Similarly, adaptation and adjustment were commonly used. Beyond the association of these terms with motherhood as a period of transition, their use may also reflect the shifting focus of resilience research from absence of psychopathology to ‘positive adaptation’, which features in several resilience definitions [108]. Pragmatic and logical analysis demonstrated that resilience was operationalised most frequently by the absence of pathological symptoms. This approach has been critiqued for framing resilience as illness absence, but resilience extends beyond ill-health; it includes variables that contribute to its development and consolidation [17] and may manifest in personal achievements, social competencies, or developmental milestones [18].

Mental health is closely tied to resilience, and measures related to mental health and well-being are commonly operationalised in resilience research [17,98]. There are relevant parallels in the discussions between mental health and its conceptualisation, and resilience and its operationalisation. The study of mental health has frequently focused on issues that may be more accurately described as mental illness or disorder, leading to mental health being presented as absence of symptomology or disease [2,109]. Similarly, conceiving resilience as illness absence neglects to provide evidence of the frequently used definitional component of the concept as a positive adaptation or function [23,110]. A predominant focus on mental illness with insufficient exploration of the well-being potential of resilience limits understanding. A closer focus on the outcomes which reflect, not only evidence of positive outcomes, but also positive outcomes that are specifically relevant to the perinatal period and early motherhood, may benefit and advance the research, in understanding what it means to be resilient in this season of life, and illuminate the domains most indicative of resilience in this context.

Several studies operationalised scales related to positive domains of functioning outside of mental well-being or ill-health; such as parental well-being [59] or postpartum sense of competence [48], which are suggested to reflect resilience in this context. These investigations were centred, mainly, on a woman’s parenting and familial role. Future research may benefit from a wider exploration of the domains in which resilience manifests, including biological, social, and cognitive functioning. Additionally, this exploration may be enhanced with insights from mothers regarding the domains or indicators that best reflect ‘positive functioning’ and resilience during this period.

At this point in time, the pragmatic application of resilience in pregnancy and motherhood has limited use in clinical practice. Beyond advice to support and assess women, there was sparse discussion as to how findings may be applied in a practical manner. The issues around varying definitional viewpoints, and the lack of agreed or consistent domains in which outcomes should be measured, restrict the pragmatic application of resilience research within this context. In addition, recommendations for screening were mainly centred on identifying risk and psychopathology, as opposed to utilising findings to develop resilience promoting programmes, interventions, or models of care.

An interesting point regarding the literature’s usefulness to research is that the findings frequently highlight the heterogeneity of women’s mental health experiences and patterns during pregnancy and following birth. The analysis demonstrates that mental health and resilience outcomes during this time are complex, and that psychological outcomes are not always negative, even in the presence of known risk factors, and are influenced by an array of nuanced social, economic, and cultural factors [44,65,74,81,83,85,86]. While this awareness helps to shape and build upon the current knowledge base, it also underlines the need for further inquiry, in order to reach a comprehensive understanding of resilience during the perinatal period and motherhood.

### Limitations

The inclusion criteria allowed for studies that did not provide a formal or operationalised definition of resilience. This may be considered a limitation, particularly in light of on-going definitional debates. However, it potentially informed a more accurate representation of the current state of literature in this context, because of the definitional debate. Researchers have routinely been invited to provide unambiguous expressions of their understanding of resilience, to avoid misunderstanding and enable other researchers to be aware of the particular framework employed by any given piece of research [108]. Researchers of resilience in the perinatal period and early motherhood should also be mindful to pre-empt such a limitation in their own research and provide clear conceptual and operational definitions of resilience.

## 5. Conclusions

This analysis consolidates the findings of resilience in the perinatal period and early motherhood; identifies reoccurring themes, limitations, and potential areas for development as they became evident through the evaluation of the philosophical principles of epistemology, linguistics, pragmatism, and logic; and offers a base on which to advance the concept in this context.

In terms of the perinatal period and early motherhood, an operationalised definition of the concept remains elusive. The study of resilience in this context allows the research focus to shift from pathogenic models, which have encapsulated this context thus far, by placing greater attention on protective mechanisms and psychosocial factors over risk and vulnerability, and positive adaptation over maladjustment. While the analysis demonstrates that this focus features within the current research, as approximately half of the studies contained positive outcomes of well-being, positive functioning, or exploration of protective factors, a continued effort needs to be maintained to ensure that future research comprehensively embraces the health orientation of the concept of resilience.

The associated concepts of coping, adaptation, and adjustment are recurring themes within the literature and may prove useful avenues of future investigation and development. In addition, exploring women’s lived experience of resilience and their perspectives on the ways in which resilience in this context has been researched to date is an important area of further inquiry.

## Data Availability

Not applicable.
